# Dengue Virus Induces NK Cell Activation through TRAIL Expression during Infection

**DOI:** 10.1155/2017/5649214

**Published:** 2017-09-05

**Authors:** Mariana Gandini, Fabienne Petitinga-Paiva, Cíntia Ferreira Marinho, Gladys Correa, Luzia Maria De Oliveira-Pinto, Luiz José de Souza, Rivaldo Venâncio Cunha, Claire Fernandes Kubelka, Elzinandes Leal de Azeredo

**Affiliations:** ^1^Viral Immunology Laboratory, Oswaldo Cruz Institute/FIOCRUZ, Rio de Janeiro, RJ, Brazil; ^2^Celular Microbiology Laboratory, Oswaldo Cruz Institute/FIOCRUZ, Rio de Janeiro, RJ, Brazil; ^3^Plantadores de Cana Hospital, Campos dos Goytacazes, RJ, Brazil; ^4^Medical Clinic Department, Federal University of Mato Grosso do Sul, Campo Grande, MS, Brazil

## Abstract

Dengue is an acute febrile illness with a wide spectrum of signs and symptoms ranging from mild to severe forms characterized by plasma leakage that can be fatal. NK cells are one of the main effectors in early infection and may play an important role in dengue pathogenesis. We investigated NK cell involvement during dengue infection. A higher frequency of NK cell subsets and TRAIL+NK cells was found in mild DF cases when compared to that in severe cases or healthy donors. NK activation markers such as CD107a and TLR3 were upregulated in patients' cells compared to those in healthy donors. In addition, IL12 related to NK cell activation were upregulated in mild DF cases. *In vitro* PBMC culture models show that DENV-stimulated and IFN*α*-stimulated NK cells were able to express TRAIL, suggesting an indirect activation of cells, regarding TRAIL expression. Type I IFN receptor blockage on DENV-stimulated PBMCs showed TRAIL expression on NK cells is partially IFN*α* dependent. In addition, during PBMC stimulation, TRAIL expression on NK cells was inversely correlated with DENV-positive monocytes. Therefore, we observed DENV-induced activation of NK cell populations. A higher activation of NK cells would promote limited viral spread, resulting in decreased inflammatory response, contributing to protection against dengue severity.

## 1. Introduction

Dengue fever is an arboviral disease endemic at tropical and subtropical regions where 2.5 billion people are at risk. No vaccine or specific treatments are currently licensed or available. Dengue is a major health problem in Brazil, responding to the majority of cases in the Americas. Dengue virus (DENV) is a flavivirus, and all serotypes (DENV-1 to 4) may cause disease in which hemorrhagic manifestations and/or effusions may lead to severe clinical forms [1]. The wide range of observed clinical forms may reflect a synergism of several causes such as host genetic factors [2–4], cross-reactive cellular and antibody responses [5, 6], and/or strain virulence [7]. However, the majority of dengue patients present only mild symptoms and recover after defervescence. Immune response to DENV may play a role in pathophysiology, in which high levels of cytokines were correlated to severity [8]. Soluble mediators released in consequence of viral infection may promote endothelial activation and, subsequently, a systemic short-term plasma leakage [1]. Besides, DENV replication may subvert innate immunity mechanisms, specially type I interferon signaling [9], suggesting a negative impact in innate immune antiviral responses.

NK cells are key players during initial viral infection, mainly acting on delaying viral spread through cytotoxicity towards infected cells. NK cells are activated by type I interferons that increase cytotoxicity against infected cells and promote immunoregulatory functions through cytokine release [10]. NK cells become activated as a result of signals received from target cells, in which the integration of signaling between NK cell membrane-bound activating or inhibitory receptors and membrane-bound ligands on infected cells dictates survival or death; activation can also indirectly result from cytokine signaling or pathogen recognition itself [11]. Effective cytotoxicity is mediated by classical degranulation, but also by expression of surface death molecules Fas (CD95/APO-1) and TRAIL (tumor necrosis factor- (TNF-) related apoptosis inducing) [12, 13]. TRAIL is a transmembrane or soluble protein of the TNF superfamily with apoptosis-inducing functions mediated by binding to its two death receptors TRAIL-R1/-R2 on target cells [14, 15]. Soluble TRAIL was antiviral against dengue [16], and its plasma levels correlated positively with mild cases, as well as IFN*α* levels [17]. Moreover, our group demonstrated that dengue infection has a positive impact on NK cell numbers during acute mild dengue disease [18]. However, NK cell function during dengue disease needs further elucidation. Considering that TRAIL expression on NK cells can be induced by type I interferons, we questioned whether NK cells could express TRAIL during dengue infection.

## 2. Material and Methods

### 2.1. Human Blood Samples

Blood from 43 dengue patients with confirmed dengue fever from two Brazilian health centers localized at Campo Grande state of Mato Grosso do Sul and Campos dos Goytacazes, state of Rio de Janeiro, was analyzed. Diagnosis of dengue cases was performed using Dengue Virus IgM Capture DxSelect™ (Focus Diagnostics, California, USA) and Platelia™ Dengue NS1 Ag ELISA (Bio-Rad Laboratories, California, USA). Molecular detection and serotype typing were performed as described previously [19]. All experimental procedures with human blood were approved by the ethical committee at Plataforma Brasil, Fiocruz (CAAE 13318113.7.0000.5248). All patients were informed of the procedures and gave written consent. Demographic information about the studied population as well as the classification criteria is described in Table 1. Blood from healthy donors for ex vivo experiments was obtained from volunteers in the state of Rio de Janeiro at Fiocruz. Hemotherapy Service, HUCFF, from Federal University of Rio de Janeiro provided buffy coats of the healthy donors.

### 2.2. Human Cell Isolation and Stimulation

Cryopreserved peripheral blood mononuclear cells (PBMC) from patients/healthy donors or fresh PBMC from healthy donors were obtained from density gradient centrifugation of either heparinized blood or buffy coats, respectively, with a Ficoll-Hypaque separation medium (GE Healthcare). *In vitro* experiments were performed with fresh PBMC from healthy donors. Cells were cultured in RPMI 1640 (Invitrogen, Gaithersburg, MD, USA) containing 10% fetal bovine serum (HyClone) and 1% penicillin-streptomycin-glutamine (Gibco) at 37°C in a humidified 5% CO2 chamber according to the protocol. Dengue virus type 2 strain Thailand/16681/1984 (DENV-2) [20] was used for viral stimulation. Viral stock preparation and titration are described elsewhere [17]. Uninfected C6/36 cell supernatant was purified and used as negative control (MOCK). PBMCs at 2 × 10^6^ cells/1.0 mL were cultured overnight with DENV-2 (MOIs 0.1, 1, or 10), 150 ng/mL of soluble recombinant IFN*α* (PBL International), MOCK, or unstimulated. When required, monoclonal antihuman IFNAR2 (PBL International) at 1.5 *μ*g/mL per well was added 60 min prior to subsequent stimulation.

### 2.3. Flow Cytometry and ELISA

Ex vivo PBMCs were immediately thawed, washed in cold wash buffer (phosphate-buffered saline, 2% fetal bovine serum, and 1 mM ethylenediamine tetraacetic acid). Cells were blocked with wash buffer containing 5% of heat-inactivated human plasma, followed by staining with CD16-FITC (SouthernBiotech), TRAIL-PE or isotype-matched antibody (BD Biosciences), CD3− Pacific Blue (BioLegend), CD107a-eFluor660 or isotype-matched antibody, and CD56-PE.Cy5.5 (eBioscience), and fixed with 2%paraformaldehyde prior to analysis. Cell cultures were stained with the same antibodies as patients' sample but CD56-PerCP.Cy5.5 (BioLegend) was used instead. For TLR3 detection, cells were further permeabilized with BD®Perm reagent and stained with TLR3-PE (BD Pharmingen). PDCs were assessed with the following antibody cocktail: CD4-PE (SouthernBiotech), CD11c-PE.Cy7 (BD Pharmingen), and BDCA4-APC (Miltenyi). About 1 × 10^5^ events in the lymphocyte gate were acquired when analyzing patients' samples in FACS Aria IIu. For *in vitro* samples, 5 × 10^5^ events were acquired in the same gate using BD Accuri C6 or FACS AriaIIu. All samples were analyzed in up to 18 hours after fixation. IL12 (R&D Systems) and IFN*α* (PBL Interferon Source) were detected in cell culture supernatant or plasma samples by ELISA.

### 2.4. Statistical Analysis

In vitro experiments were repeated at least four times. *p* values (*p*) were determined using a two-tailed Wilcoxon matched pair test for paired *in vitro* data and nonparametric Mann–Whitney test for patient data (Prism 6.0, GraphPad). *p* < 0.05 was considered statistically significant. Values were submitted to one-way analyses of variance to test for linear trend in which *p* < 0.05 was statistically significant.

## 3. Results

### 3.1. NK Cells Display Increased Frequency in Mild Dengue Patients

The patients enrolled in the study presented fever accompanied by one or more of the following symptoms: myalgia, arthralgia, exanthema, headache, prostration, pruritus, conjunctival hyperemia, edema, nausea, vomiting, and retro orbital pain. Dengue-infected patients were classified according to the latest WHO classification [21]. Of these, 28 were classified as DF without warning signs, 5 as DF with warning signs, and 10 as severe dengue. There were no significant statistical differences in age, sex, fever, or other symptoms between groups of patients analyzed. However, severe patients presented lower platelet counts and higher ALT levels as compared with DF without warning signs/DF with warning signs. Severe cases had a higher frequency of persistent abdominal pain, followed by uncontrollable vomiting and strokes (pericardial or pleural or ascites). Demographic and laboratorial information are summarized in Table 1.

**Figure 1:**
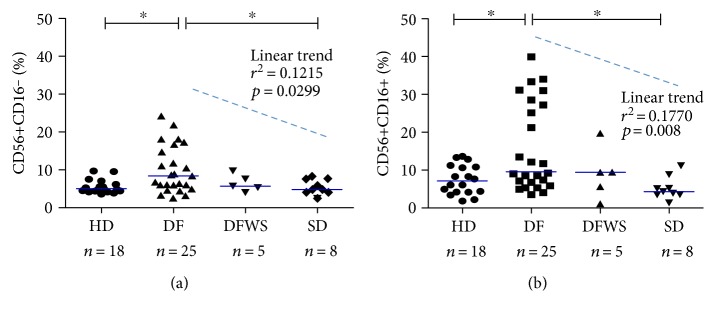
NK cell populations' frequency among dengue-infected patients. Patients' PBMCs collected during disease acute phase were phenotyped by CD56/CD16 cell surface density analysis by flow cytometry. Graphs represent median values (blue line) for NK subpopulation CD56+CD16− (a) or CD56+CD16+ (b) frequencies among total PBMCs from patients or healthy donors (HD) in which each point represents one subject. Patients were ranked by severity: Dengue fever without warning signs (DF) or with warning signs (DFWS) and severe dengue (SD). Values were submitted to Mann–Whitney statistical test (for comparison between two groups in which^∗^*p* < 0.05) and one-way analysis of variance for linear tendency between group severity and analyzed parameters.

NK cells exert their function during viral infection mainly by cytotoxicity towards infected cells and through cytokine production [22]. Because CD16+ NK cells are considered highly cytotoxic and CD16− NK cells feature enhanced cytokine production, we analyzed the frequencies of CD56+CD16− and CD56+CD16+ subsets among NK cells during acute dengue disease (Figure 1). Both subsets show frequencies among PBMCs greater in dengue fever patients (DF) than those from NK cells of healthy donors (HD) or severe dengue (SD) cases. Patients presenting warning signs (DFWS) displayed intermediate percentages of both NK cell subsets, suggesting an association between NK cell circulating frequencies and disease severity. Indeed, we observed a negative linear trend towards severity when CD56+CD16− NK cells are analyzed (linear trend: slope −2.41, *r*^2^ 0.1215, *p* = 0.0299). Therefore, dengue infection has a positive impact on NK cell population.

### 3.2. Dengue Patients' NK Cells Display Activation Features: CD107a and TLR3 Expression Are Upregulated and TRAIL Is Increased in Mild Patients

**Figure 2:**
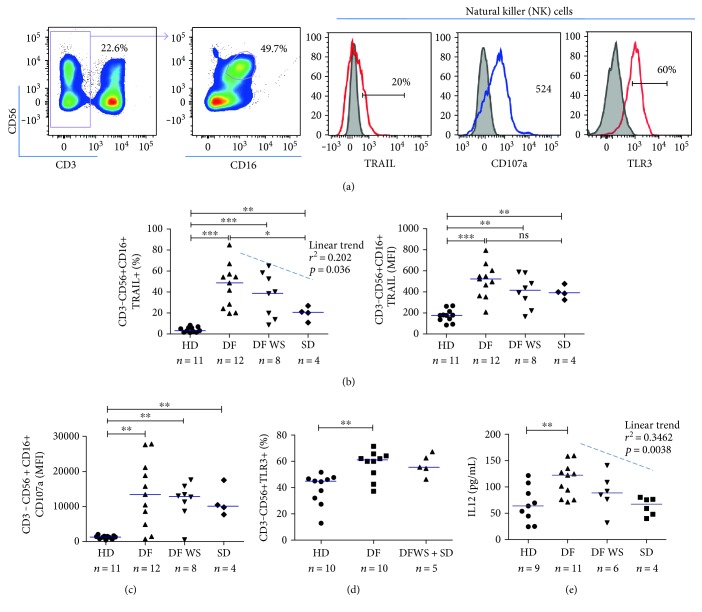
NK cell activation profile in dengue-infected patients' samples. Patients' PBMCs collected during disease acute phase or healthy donors PBMCs were thawed and immediately phenotyped by CD3/CD56/CD16/TRAIL or TLR3/CD107a cell surface density analysis by flow cytometry. IL12 levels were detected in plasma samples by ELISA. Analyzed cells (a) were gated on CD3− cells and then TRAIL-positive cells or TRAIL MFI (b) and CD107a median (c) were analyzed on CD3−CD56+CD16+ NK cells. TLR3-positive cells (d) were detected in all CD56+ cells. IL12 levels (e) were detected at acute phase samples or healthy control samples. Subjects were grouped into healthy donors (HD) or patients with dengue fever without warning signs (DF), dengue fever with warning signs (DFWS), or severe dengue (SD). Values were submitted to Mann–Whitney statistical test (for comparison between two groups in which ^∗^*p* < 0.05, ^∗∗^*p* < 0.005 and ^∗∗∗^*p* < 0.0005) and one- way analysis of variance for linear tendency between group severity and analyzed parameters.

To study NK cell activation/cytotoxic profile during dengue infection, apoptotic inducer TRAIL, membrane degranulation marker CD107a [23], and pattern recognition TLR3 were assessed in patients' CD3−CD56+CD16+ NK cells. Because frequencies of CD16− NK cells were low, we elected only CD16+ NK cells for that analysis, as gated in Figure 2(a).

TRAIL+ NK cell frequency and TRAIL MFI on NK cells were significantly higher in PBMC during acute dengue fever as compared to those in healthy donors (Figure 2(b)), as well as CD107a expression (Figure 2(c)) and TLR3-positive NK cells (Figure 2(d)). Remarkably, TRAIL+ NK cell frequency was significantly greater in PBMC of mild cases of dengue (DF) as compared to that in severe cases (SD) (Figure 2(b)); however, the same was not observed for TRAIL MFI analysis in the same population. Moreover, a significant negative linear trend towards severity was observed considering TRAIL-positive cells on CD3−CD56+CD16+ NK cells from patients (linear trend: slope −12,83, *r*^2^ 0.2016, *p* = 0.0359). We observed no difference in CD107a expression or TLR3-positive cells between disease groups studied.

Circulating cytokines may promote NK cell activation and therefore were analyzed in plasma samples of those patients. Previous work from our group described a significantly enhanced production of IFN*α* and soluble TRAIL in plasma samples from the same patient group analyzed in this work [17] and was significantly more pronounced in mild patients as compared to that in severe ones. IL12 can both promote IFN*γ* production and TRAIL expression by NK cells [24, 25]. When soluble IL12 was analyzed in plasma samples, significant higher levels were found in mild dengue (DF) patients (median 122 pg/mL, *n* = 11, *p* = 0.006) as compared to that in healthy donors (median 63 pg/mL, *n* = 9) and a negative trend toward severity was observed (Figure 2(e)). Taken together, we observed that the degree of NK cell activation may have a role in disease progression.

### 3.3. NK Cells Express Membrane TRAIL after *In Vitro* DENV Stimulation

**Figure 3:**
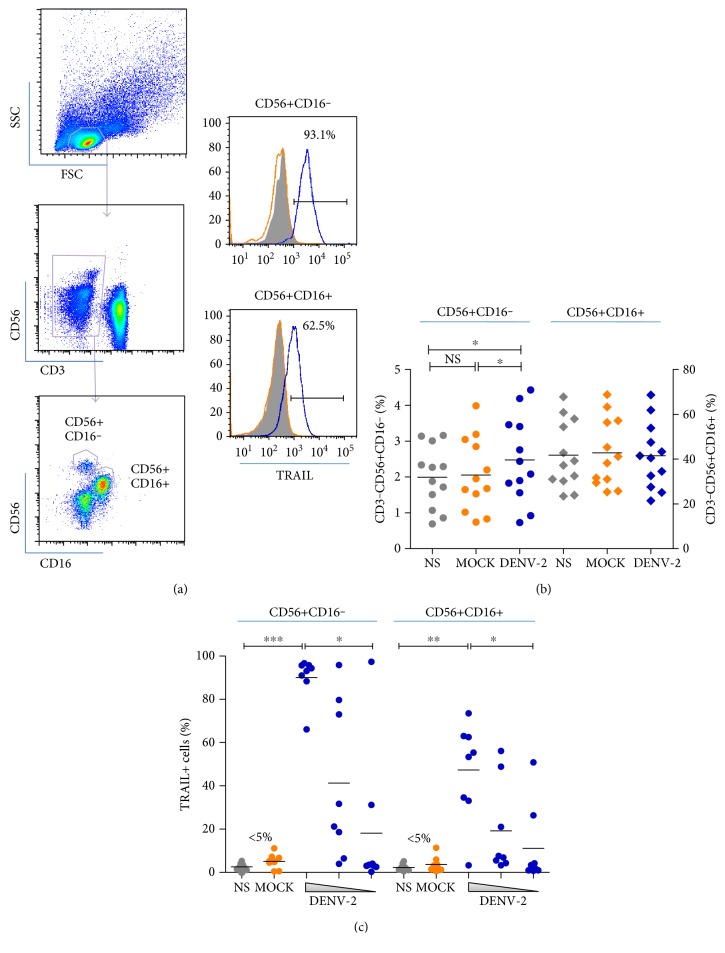
DENV-2-stimulated NK cells: subpopulation frequencies and TRAIL expression analysis. Healthy donors' PBMCs were freshly obtained and subsequently stimulated with DENV-2 at several multiplicities of infection (10, 1, and 0.1) or negative controls as MOCK (uninfected C636 cell culture supernatant) or not stimulated (NS) for 18 hours. (a) NK cells were gated as CD3−CD56+CD16+ or CD3−CD56+CD16− and TRAIL-positive cells were detected for each subpopulation. (b) CD3−CD56+CD16− (left) or CD3−CD56+CD16+ (right) frequencies among PBMCs stimulated with MOCK (orange), DENV-2 (blue) or not stimulated (gray) are shown. (c) TRAIL-positive cells among CD3−CD56+CD16+ or CD3−CD56+CD16− after stimulation with DENV-2, MOCK, or not stimulated. Values were submitted to Friedman's test with Dunn's multiple comparison test in which differences with *p* < 0.05 (∗), *p* < 0.005 (∗∗) or *p* < 0.0005 (∗∗∗) were considered statistically significant.

In order to understand TRAIL expression on NK cells during DENV infection, we investigated whether *in vitro* DENV stimulation could enhance membrane TRAIL detection on NK cells. Both CD3−CD56+CD16− and CD3−CD56+CD16+ NK cell subsets were analyzed (Figure 3(a)). Overnight stimulation of the healthy donors' PBMCs with DENV-2 significantly impacted on CD16− NK cell frequency as compared to mock infection or unstimulated cells (*n* = 12, *p* < 0.05). No differences were detected for CD16+ NK cell frequency after DENV stimulation. Membrane TRAIL expression was observed NK cells in both subsets as compared to mock-treated or unstimulated NK cells (Figure 3(c)). TRAIL detection was DENV-2 dose dependent on both subsets of NK cells analyzed as evidenced by a decreased TRAIL detection when lower MOIs were tested (Figure 3(c)). Remarkably, CD3−CD56+CD16− NK cells exhibit more TRAIL+ cells than CD16+ subpopulation once compared at higher DENV-2 MOIs (*n* = 12, *p* < 0.05). Therefore, we observed that dengue infection is able to induce TRAIL expression on NK cells during both ex vivo and *in vitro* studies.

### 3.4. Type I Interferons Are Involved in TRAIL Expression on NK Cells during DENV-2 Stimulation

**Figure 4:**
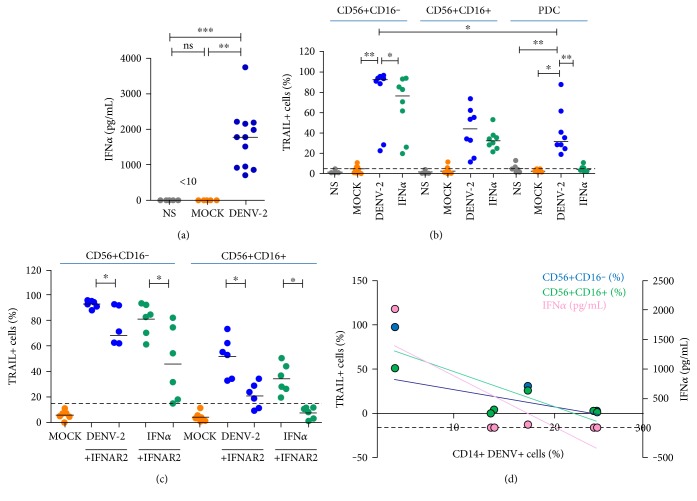
Type I interferon involvement in TRAIL expression on NK cells. Healthy donors' PBMCs were freshly obtained and subsequently stimulated with DENV-2 at MOI 10, recombinant IFN*α* (150 ng/mL) or negative controls as MOCK (uninfected C636 cell culture supernatant), or not stimulated (NS) for 18 hours. Type I interferon subunit receptor 2 (IFNAR2) was blocked by treating cells with 1.5 *μ*g/mL of IFNAR2-neutralizing antibody 30 min prior to viral stimulation. IFN*α* was detected in the cell culture supernatant by ELISA. (a) IFN*α* production by PBMC cultured with DENV-2 (blue) or MOCK (orange) in comparison with basal levels (gray). (b) Recombinant IFN*α* (green), mock-stimulated (orange), DENV-2-stimulated (blue), or unstimulated (grey) CD56+CD16− NK cells (left), CD56+CD16+ NK cells (center) or plasmacytoid dendritic cells (PDC—right) positive for membrane TRAIL. (c) Recombinant IFN*α* or DENV-2-stimulated CD56+CD16− (left) or CD56+CD16+ (right) TRAIL+ NK cells treated or not with IFNAR2 neutralizing antibody. (d) Correlation between MOI 0.1 DENV-stimulated TRAIL+ NK cells (left *y* axis: CD16− as blue line and CD16+ as green line) or IFN*α* detected in supernatant (right *y* axis: pink) and CD14+ DENV+ monocytes among PBMCs stimulated with DENV-2 MOI10. Values were submitted to Friedman's test with Dunn's multiple comparison (a) in which differences with *p* < 0.05 (∗), *p* < 0.005 (∗∗) or *p* < 0.0005 (∗∗∗) were considered statistically significant. Comparisons between two groups of data (b and c) were submitted to Wilcoxon's matched pairs test.

Considering that type I interferons mediate TRAIL expression on NK cells [12], we next sought to study a role for those cytokines during *in vitro* stimulation. Firstly, we observed that IFN*α* was detected in the supernatant only during DENV stimulation (*n* = 12, *p* < 0.05) compared to mock or unstimulated cells (Figure 4(a)). Then, we observed that TRAIL+ NK cells were detected after overnight PBMC stimulation with recombinant IFN*α* (Figure 4(b)). We also observed that the cytokine had a higher capacity of inducing TRAIL in CD16− NK cells as compared to CD16+ NK cells during stimulation. However, it was not possible to reproduce the high frequency of DENV-stimulated TRAIL+NK cell with recombinant IFN*α*, despite that higher levels of the cytokine were found in IFN*α*-stimulated supernatants (3309 ± 715.7 pg/mL) as compared to DENV-stimulated supernatants (1642 ± 195.8 pg/mL; *n* = 8; *p* = 0.04). Moreover, we observed that TLR3 expression on CD16− NK cells was significantly enhanced (MFI 3404± *n* = 3) compared to that in MOCK (MFI 2924 ± 249.4, *p* = 0.002) or in unstimulated cells (data not shown). Recently, we demonstrated that PDCs only express TRAIL when DENV viral particles are internalized [17]. Indeed, IFN*α*-stimulated PDCs were not able to express TRAIL as opposed to DENV-stimulated PDCs (Figure 4(b)). This suggests that, in opposition to PDCs, induction of TRAIL expression on NK cells may account for cytokine stimulation rather than viral particle internalization.

To further explore the role of type I interferons in TRAIL expression, we neutralized interferon alpha receptor 2 (IFNAR2) with the aid of monoclonal antibodies before viral stimulation (Figure 4(c)). Recombinant IFN*α* was tested to assess blocking efficiency. Treatment was efficient in blocking IFN-stimulated TRAIL expression on CD16+ NK cells (78.2% inhibition, *n* = 6, *p* < 0.05); however, IFNAR2 blocking allowed only a significant 46.5% reduction in TRAIL expression on CD16− NK cells. DENV-stimulated NK cells also presented diminished TRAIL expression when IFNAR2 was blocked. However, CD16+ NK cells are apparently more sensitive to IFNAR2 blocking than CD16− NK cells, because a reduction of 43.9% was observed in TRAIL expression of CD16+ NK cells as compared to 26.3% reduction in CD16+ NK cells. We observed that IFNAR blocking had no significant impact on IL12 levels during *in vitro* DENV stimulation (DENV only: median 86.17 ± 53.27 pg/mL as compared to DENV plus IFNAR: 161.58 ± 55.26 pg/mL; *n* = 3). Recombinant IFN*α* stimulation (with or without IFNAR blockage; less than 7 pg/mL; *n* = 3), mock stimulation (12.19 ± 3.4 pg/mL), or unstimulated (17.4 ± 2 pg/mL) had low impact on IL12 levels. Finally, we correlated the maximal dengue antigen detection on monocytes (MOI 10) among PBMC to minimal production/expression of IFN*α* or TRAIL+ NK cells, respectively, to observe how fast donors can respond to DENV as compared to how much DENV can replicate.  Figure 4(d) demonstrates borderline significant inverse correlations concerning viral antigen detection, CD16− TRAIL+ NK cells (Pearson's *r* = −0.7687, *p* = 0.0740), CD16+ TRAIL+ NK cells (Pearson's *r* = −0.7339, *p* = 0.0968), and IFN*α* detection on supernatant of MOI 0.1 DENV-stimulated PBMCs (Spearman's *r* = −0.8286, *p* = 0.0583). Taken together, these data account for an important but not exclusive bystander activation of NK cells by type I interferons and DENV.

## 4. Discussion

Dengue fever is a self-limiting febrile illness in which a life-lasting immunity is reported, even at severe cases [1]. Therefore, innate immune response deregulation may be crucial to disease outcome. Because virus loads decrease significantly over time of dengue infection, it is believed that immune response is effective in controlling viral burden, though at a cost of an enhanced inflammatory response to the host. NK cells are endowed with two effector roles: cytotoxicity towards infected cells and cytokine production, mainly IFN*γ*, therefore contributing to both viral clearance and inflammatory cytokine production [26].

We observed here that DENV infection influences NK cell frequencies in both subpopulations analyzed. These data are in line with our previous report [18] and also with previous publications in which a positive correlation between circulating NK cells and disease prognosis is reported [27, 28], suggesting that dengue infection has an impact on NK cell frequencies. Indeed, an initial augmentation on NK cell frequencies was also reported for other viral illnesses. For instance, yellow fever-vaccinated volunteers display enhanced frequencies of CD56+CD16+ NK cells seven days after vaccination [29]; and an early expansion followed by contraction was observed for the frequencies of NK cells from EBV-infected individuals [30]. Efficient antiviral responses rely on sufficient cell numbers, and fluctuations may reflect a higher production by bone marrow precursors, redistribution in infected tissues, or even NK cell expansion in the periphery. Indeed, IL15 levels found during acute dengue [18] may promote NK cell subset expansion. Moreover, during *in vitro* assays, we observed that DENV-2 stimulation could alter NK cell CD16− frequency and cytokines such as IFN*α* and IL12 found in supernatants. Possibly, cytokines in the milieu might act promoting cell expansion, especially on CD16− NK cells, once they are highly responsive to cytokines. Therefore, we suggest that NK cell frequencies may be correlated to protection during dengue disease.

However, NK cell numbers may not represent NK cell activation status, as it was observed for elderly individuals [30], making activation/cytotoxic phenotype an important object of analysis. We report here that CD16+ NK cell subpopulations are more likely to be activated in mild cases of dengue, when TRAIL, CD107a, and TLR3 were analyzed. Our group also reported other activation markers such as CD69 and TIA1 as upregulated in mild dengue patients [18]. However, some groups suggest that NK cell activation may have a detrimental role during dengue disease. CD69+ NK cells were correlated with severe [27] and with shock syndrome cases [31]. Despite that, recently, several genes correlated with NK cell activation were observed less abundantly in shock syndrome patients [32] and some might predict mild over severe cases [33]. Moreover, TRAIL expressed on NK cells was associated to a decreased viral load, specially during hepatitis C virus treatment with type I interferon [34] or during acute accidental HCV-controlled infection [35]. TRAIL expression seems to be important in other viral infections such as WNV and influenza murine models, in which TRAIL−/− mice fail to control viral replication [36, 37]. Taken together, these data reinforce the cytotoxic role of TRAIL during viral infections. CD107a is a marker of degranulation of cytolytic granules [23], and its expression protects NK cells against their own cytotoxicity [38]. Although CD107a was not correlated with severity, we observed it might indicate NK cell cytotoxicity towards dengue infection. Indeed, CD107a expression was correlated to NK cell activation in HCV [39], influenza [40], or HIV [41] infections. Accordingly, CD4 and CD8 T cells expressed CD107a during dengue; however, no association to severity was found [42]. Like CD107a expression, TLR3 expression was not different among dengue severity, but segregated healthy donors and mild patients. The importance of TLR3 in the induction of an antiviral response against flavivirus infections has been demonstrated. TLR3 can bind DENV antigens triggering IFN*α*/*β* antiviral responses [43, 44]. More recently, it was demonstrated that ZIKV-infected fibroblasts expressed TLR3 and TLR3 inhibition, resulting in a strong increase in viral replication [45]. Albeit a role of TLR3 in NK cells during viral infections is still elusive, yellow fever vaccination was able to enhance detection of TLR3 and TLR9 in NK cells early after vaccination [29], suggesting its role as an activation marker for NK cells. Indeed, TLR3-agonist poli I:C activation promoted *in vitro* CD69 expression on NK cells [46] and a low TRAIL expression [47]; viral stimulation is also able to upregulate TLR3 expression [48].

The role of IL12 during dengue infection is still elusive, however implicated in severity [49] or as a good outcome marker [50]. Regarding NK cells, not only target cells but also cytokines can activate this cell type, in which IL12 is a classic cofactor for IFN*γ* production [25]. Even though IFN*γ* plasma levels were correlated with dengue severity [11], in our work, IL12 levels were enhanced in mild cases. Because IL12 is involved in human NK cell cytotoxicity and alone can upregulate the activation marker NKG2D, as well as TRAIL expression on the NK cell surface [30, 31], we suggest that IL12 may have a protective role regarding this cell type.

Membrane TRAIL expression on NK cells is sufficient to promote cytotoxicity as reported elsewhere [34, 51, 52], suggesting this cytotoxic mechanism could be active also during dengue disease. DENV infection upregulates MHC I expression on infected cells [53], and dengue-specific antibody dependent cell cytotoxicity was required for *in vitro* NK cell-mediated cell death [54], suggesting that dengue could block NK activation. TRAIL was differentially expressed in NK cell subpopulations analyzed here, and this dichotomy may reflect different cytotoxicity features between these subsets. CD56^bright^ (here CD56+) CD16− NK cells express very low levels of perforin and depend on TRAIL for their cytotoxicity [55]. This subset is abundant in secondary lymphoid organs (SLO) where immunoregulatory functions are observed through cytokine production [56]. TRAIL+CD56+CD16− could promote apoptosis of super stimulated or infected cells localized in SLO, diminishing inflammatory response and viral load. Moreover, because dengue-infected antigen-presenting cells migrate to SLO in theory, NK cell-mediated cytotoxicity towards this population may promote effective T cell stimulation, since dengue infection disturbs APC maturation [57]. TRAIL appears to be able to induce apoptosis in some DENV-infected cells as reported previously [58]. Additionally, cultured monocytes from dengue-infected patients produced high levels of TRAIL and showed high expression of apoptosis [59]. On the other hand, some studies did not find apoptosis of DENV-infected monocytes through TRAIL expression [16, 60]. It remains unclear whether TRAIL+NK cells could induce apoptosis of dengue-infected cells.

Type I interferons are able to upregulate TRAIL on NK cells [12], though NK subsets respond differently to cytokine stimulation. We were unable to completely abrogate TRAIL expression on CD16− NK cells by IFNAR blocking, especially with DENV-2 stimulation. Apparently, this difference may reflect IFNAR density on cell surface, TRAIL signaling in response to another cytokine, such as IL12, or even STAT1/4 available to IFNAR [10]. Although other factors may be involved, we observed that type I interferons are major contributors to *in vitro* TRAIL induction in NK cells during DENV-2 stimulation. Moreover, recently, *in vitro* NK cell-mediated cytotoxicity was enhanced by type I interferon treatment in a model of NK-infected DC coculture [61], suggesting an important crosstalk between APCs and NK cells for viral clearance. For instance, HCV patients' NK cells exhibited low levels of degranulation and TRAIL expression *in vitro*, which were recovered by IFN*α* [62]. Our group observed that IFN*α* and PDC activation were enhanced in mild dengue patients, and here we observed NK cell activation was also enhanced in the same patient group [17]. We also observed here that donor permissiveness to dengue replication correlates inversely with TRAIL+ NK cells and also with IFN*α* production. It was already reported that soluble TRAIL [16] and IFN*α* [17] can reduce DENV replication in human monocytes, suggesting NK cells might participate directly in a strong innate immune response against acute DENV infection.

These data strongly support that PDC-NK cell crosstalk during dengue would be beneficial to the host. A fast and productive type I interferon secretion by activated PDCs would enhance NK cell cytotoxicity towards infected cells, leading to a fast viral clearance. Although there are many questions about its function, we described here a small part of a possible antiviral mechanism of innate response during DENV infection that can be useful for the development of new drugs or other interventions as well as in the understanding of dengue pathogenesis.

## Figures and Tables

**Table 1:** Demographic, clinical, and laboratorial characteristics of DENV infected patients.

Characteristics	DF & DFWS^2^	(*N*)	Severe dengue	(*N*)^3^
Age (median, 25–75%)	43, 26–58	(33)	42, 24–50	(10)
Gender (M : F)	14 : 19	(31)	5 : 5	(10)
Infecting serotype				
DENV-2 positive/tested%	9/33 (27.2%)	(33)	1/10 (10.0%)	(10)
DENV-1 positive/tested%	4/33 (12.1%)	(33)	0/10 (0%)	(10)
Previous dengue infection (IgG positive)	79%	(30)	100%	(8)
Platelet count (×10^3^/mm^3^)^4^	121 (82–146.8)	(23)	28 (2.5–77.4) +^5^	(7)
Hematocrit	42 (39.8–43.6)	(22)	43 (38.4–47)	(7)
Leucocyte count (×10^3^/mm^3^)	4500 (3442–6218)	(27)	2750 (1737–4270)	(8)
ALT (IU/L)	63 (47.6–140.1)	(23)	159 (54–583) +^5^	(8)
AST (IU/L)	74.5 (56.8–132)	(23)	145 (115.7–667.7)*^δ^*	(9)

Study population *n* = 43; ^2^DF (dengue fever without warning signs) and DFWS (dengue fever with warning signs; severe dengue according to [21]); ^3^number of patients with available data; ^4^C.I., 95% confidence interval; ^5^*+p* < 0.05 represents statistical difference of DF and DFWS versus severe dengue; Mann–Whitney nonparametric test was applied; *^δ^p* = 0.0945.
